# The complete chloroplast genome of *Lagerstroemia loudonii* Teijsm. & Binn. (Lythraceae), an ornamental tree with medicinal value

**DOI:** 10.1080/23802359.2022.2093671

**Published:** 2022-07-06

**Authors:** Bo Qin, Kaidao Sun, Xin Huang

**Affiliations:** Guangxi Key Laboratory of Special Non-wood Forest Cultivation & Utilization, Guangxi Forestry Research Institute, Nanning, China

**Keywords:** Chloroplast genome, *Lagerstroemia loudonii*, phylogenetic analysis

## Abstract

*Lagerstroemia loudonii* is an ornamental tree with medicinal value. Here, we announce the first complete chloroplast genome sequence of *L. loudonii.* The *L. loudonii* chloroplast genome harbors a typical quadripartite structure with a total length of 152,372 bp, including a large single-copy (LSC) region of 84,086 bp, a small single-copy (SSC) region of 16,798 bp, and two separated inverted repeat (IR) regions of 25,744 bp each. The chloroplast genome encodes 130 genes, including 85 protein-coding genes, 37 transfer RNA (tRNA) genes, and 8 ribosomal RNA (rRNA) genes. The GC content of the whole chloroplast genome is 37.6%. Phylogenetic analysis based on complete chloroplast genomes of *L. loudonii* and 17 other plant species revealed that *Lagerstroemia* is a separate genus, and *L. loudonii* is closely related to *L. calyculata.*

*Lagerstroemia* Linn. 1759 belonging to the Lythraceae family is a genus with great economic value and comprises about 55 species distributed worldwide (Furtado and Srisuko 1969; Gu 2016). *Lagerstroemia* species have been widely used as ornamental plants due to their long flowering period, rich flower colors, and strong stress resistance to inferior habitats (Wang et al. 2011). *Lagerstroemia loudonii* Teijsm. & Binn. 1863 is a deciduous tree up to about 20 m tall, which is mainly distributed in Cambodia, Laos, and Thailandia (Plants of the World Online). The flowers of *L. loudonii* are 6–7 cm in diameter and are of purple color in the beginning and then turn white with development. *L. loudonii* blooms from February to April, which is significantly earlier than most of the other *Lagerstroemia* species that bloom from May to September. In addition to the high ornamental value, *L. loudonii* also has medicinal value, with its leaves being used in traditional medicine to treat diabetes, inflammatory, and obesity in East Asia (Riyanti et al. 2020). At present, the research of *L. loudonii* is mainly focused on chemical constituents and pharmacology. However, there are few reports about classification and evolution of *L. loudonii*. In this study, we assembled the first complete chloroplast genome sequence of *L. loudonii* and analyzed the characters of the complete chloroplast genome sequence and phylogenetic relationship of *L. loudonii*, which will contribute to the future studies on its evolution, genetics research and classification.

Fresh leaves of *L. loudonii*, which was previously introduced from Thailandia, were sampled from Nanning, Guangxi, China (22°53'N, 108°20'E). Voucher specimens were preserved at the herbarium of Guangxi Forestry Research Institute (http://www.gxlky.com.cn/, Mr. Li, email: zzcx_gfri@163.com) under the registration number of 2021122003. DNA samples were stored at Guangxi Key Laboratory of Special Non-wood Forest Cultivation and Utilization (Nanning, China). There was no endangered or protected species involved in the study, and no specific permissions from the authority were required for the sample. The plant is located in a residential area, and we got the permission from the managers before collecting the sample. Total genomic DNA was extracted following the method proposed by Doyle (1987). DNA libraries were constructed with an average length of 350 bp by using the NexteraXT DNA Library Preparation Kit (Illumina, San Diego, CA) and then sequenced on an Illumina NovaSeq 6000 platform. Raw sequence reads were subsequently filtered utilizing the NGS QC Toolkit (Patel and Jain 2012), and the *de novo* assembly process was accomplished by using SPAdes v3.11.0 software (Bankevich et al. 2012). Finally, the chloroplast genome was annotated by using PGA (Qu et al. 2019) and submitted to the GenBank under the accession number of NC_061954.

The *L. loudonii* chloroplast genome is 152,372 bp in size with a GC content of 37.6%. It consists of a pair of inverted repeat (IR) regions of 25,744 bp each, separated by a large single-copy (LSC) region and a small single-copy (SSC) region of 84,086 and 16,798 bp, respectively. The whole *L. loudonii* chloroplast genome harbors 130 genes, including 85 protein-coding genes, 37 transfer RNA (tRNA) genes, and 8 ribosomal RNA (rRNA) genes.

We used Blastp (https://blast.ncbi.nlm.nih.gov/Blast.cgi) to select sixty-six homologous protein-coding genes in each of other 17 species with *L. loudonii* chloroplast from NCBI, then we choose *Punica granatum* (NC_035240.1) from different family and used it as outgroup. We aligned them by using MAFFT 7.037 (Katoh and Standley 2016) with strategy of FFT-NS-2. And we used model finder to select TVM + F + I + G4 model (Kalyaanamoorthy et al. 2017) and constructed the phylogenomic tree by IQtree 2.0 (Minh et al. 2020) with 1000 bootstrap and maximum likelihood method. The results of the phylogenetic analysis proposed that *Lagerstroemia* is a separate genus and all the examined *Lagerstroemia* species were divided into three clades. *L balansae*, *L. tomentosa*, *L. loudonii*, *L. calyculata* and *L. floribunda* formed a monophyletic clade and *L. loudonii* has a close relationship with *L. calyculata* (Figure 1).

**Figure 1.**
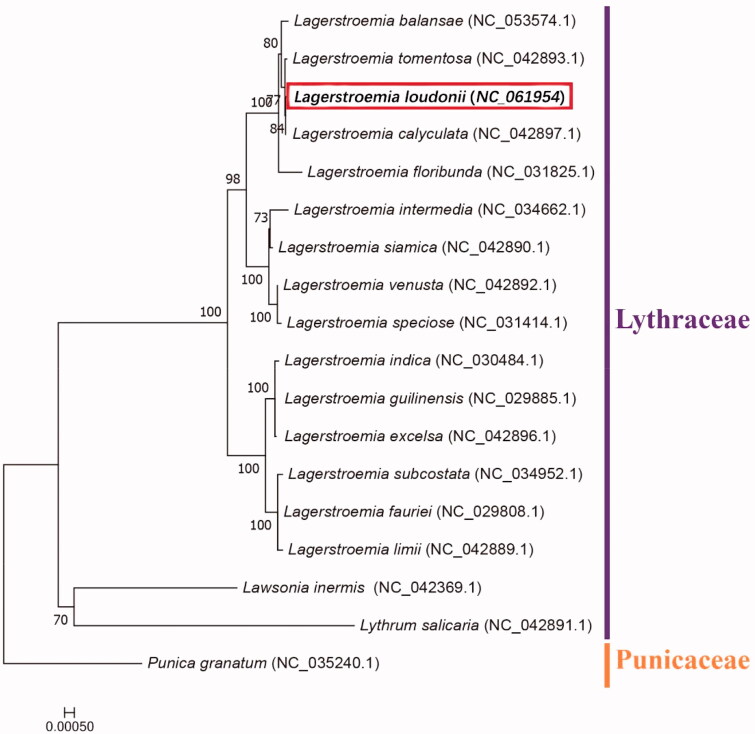
The maximum likelihood (ML) phylogenetic tree constructed based on the complete chloroplast genomes of *L. loudonii* and 17 other species, with *Lawsonia inermis*, *Cuphea hookeriana,* and *Punica granatum* as the outgroups. Numbers near the nodes represent the ML bootstrap values.

## Data Availability

The genome sequence data that support the findings of this study are openly available in GenBank of the NCBI under the accession number of NC_061954. The associated BioProject, SRA, and Bio-Sample numbers are PRJNA791531, SRS11377984, and SAMN24292890, respectively.
